# Oxidative Stress in Children with Chronic Spontaneous Urticaria

**DOI:** 10.1155/2016/3831071

**Published:** 2016-04-03

**Authors:** Fatih Dilek, Deniz Ozceker, Emin Ozkaya, Nermin Guler, Zeynep Tamay, Siddika Kesgin, Mebrure Yazici, Abdurrahim Kocyigit

**Affiliations:** ^1^Department of Pediatric Allergy and Immunology, Bezmialem Vakif University Medical Faculty, Adnan Menderes Bulvari, Vatan Caddesi, Fatih, 34093 Istanbul, Turkey; ^2^Department of Pediatric Allergy and Immunology, Istanbul University Istanbul Medical Faculty, Fatih, Capa, 34093 Istanbul, Turkey; ^3^Department of Pediatrics, Istanbul Bilim University, Buyukdere Caddesi No. 120, Esentepe, Sisli, 34394 Istanbul, Turkey; ^4^Department of Clinical Biochemistry, Bezmialem Vakif University Medical Faculty, Adnan Menderes Bulvari, Vatan Caddesi, Fatih, 34093 Istanbul, Turkey

## Abstract

The pathogenesis of chronic spontaneous urticaria (CSU) has not been fully understood; nevertheless, significant progress has been achieved in recent years. The aim of this study was to investigate the possible role of reactive oxygen species (ROS) in the pathogenesis of CSU. Sixty-two children with CSU and 41 healthy control subjects were enrolled in the study. An extensive evaluation of demographic and clinical features was done, and serum oxidative stress was evaluated by plasma total oxidant status (TOS) and total antioxidant status (TAS) measurements. The median value of plasma TOS was found to be 10.49 *μ*mol H_2_O_2_ equiv./L (interquartile range, 7.29–17.65) in CSU patients and 7.68 *μ*mol H_2_O_2_ equiv./L (5.95–10.39) in the control group. The difference between the groups was statistically significant (*p* = 0.003). Likewise, the median plasma TAS level in the CSU group was decreased significantly compared to that of the control group (2.64 [2.30–2.74] versus 2.76 [2.65–2.86] mmol Trolox equiv./L, resp., *p* = 0,001). Our results indicated that plasma oxidative stress is increased in children with CSU when compared to healthy subjects, and plasma oxidative stress markers are positively correlated with disease activity.

## 1. Introduction

Chronic urticaria (CU) is defined as urticaria that has been continuously or intermittently present for at least six weeks [1]. The lifetime prevalence of CU is 2-3% in the general population, and at any time 0.5–1% of the population suffers from the disease [1, 2]. The prevalence of CU in children is much lower than in adults and is reportedly as low as 0.1–0.3% of the child population [3]. The etiology of CU is quite heterogeneous; autoimmunity, physical stimuli, infections, vasculitis, and allergies (e.g., foods, drugs, latex, and food constituents) are major etiologic causes [1]. An underlying cause can be outlined in only 20–55% of children with CU [4, 5]. Physical stimuli are the most commonly identified etiological reasons, and approximately 15% of patients with CU have a physical trigger for the development of urticarial lesions; this subgroup of CU is termed as physical urticaria (PU) [6].

The exact pathogenesis of CU is not well delineated. The presence of persistent activation of dermal mast cells is a hallmark of CU pathogenesis, but the underlying mechanism(s) of mast cell activation is/are an enigma [1]. Autoimmune origin has become the most accepted hypothesis in recent years [7]. Functional autoantibodies in CU patients' sera have been demonstrated against IgE and Fc*ε*RI*α* by basophil and mast cell histamine release assays, by basophil activation assays, and by autologous serum skin test (ASST) [8]. This subgroup comprises roughly one-third of CU patients and is called autoimmune urticaria (AIU) [2, 9]. Chronic spontaneous urticaria (CSU) is by far the most common form of CU. In this form, triggering factors cannot be revealed despite detailed clinical and laboratory investigations.

Reactive oxygen species (ROS) are defined as molecules containing oxygen with unpaired electrons capable of initiating oxidation [11]. Although ROS play roles in host defense (e.g., phagocytosis) and as messenger molecules of the autocrine and paracrine systems, overproduction of these molecules can cause tissue damage and inflammation and even cell death [12, 13]. Fortunately, our bodies are equipped with an effective antioxidant system that includes enzymes, proteins, and low-weight molecules [12]. All inflammatory cells produce a significant amount of ROS upon stimulation [11]. Eosinophils have elevated peroxidase levels compared to other inflammatory cells and play a unique role in generating oxidative stress [11]. Also, mast cell stimulation via Fc*ε*RI signaling causes intracellular and extracellular ROS generation [14–16]. Blockades of ROS generation in mast cells by a pharmacological inhibitor cause a decrease in the release of preformed granular mediators [17]. There is a growing body of literature indicating that ROS can cause endothelial dysfunction and increased vascular permeability [18, 19]. In the light of the above information we aimed to investigate possible role of ROS in the pathogenesis of CSU.

## 2. Materials and Methods

### 2.1. Participants

**Figure 1:**
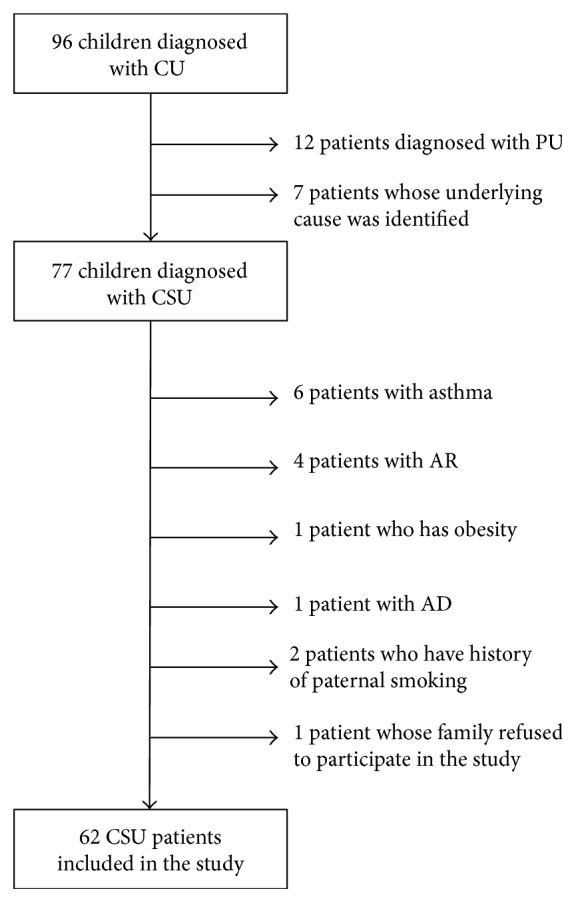
Flow diagram of patients with CU. Exclusion criteria are shown to the right. CU, chronic urticaria; CSU, chronic spontaneous urticaria; PU, physical urticaria; AR, allergic rhinitis; AD, atopic dermatitis.

All patients (total of 96) who were admitted with an urticarial episode lasting more than six weeks between February and December 2014 to pediatric allergy outpatient clinics of two university hospitals (Istanbul University Istanbul Medical Faculty and Bezmialem Vakif University) were enrolled in the study. Detailed medical histories were taken and physical examinations were performed. Subjects who had acute infections, who had a history of maternal or paternal smoking, who were taking any medications except antihistamines or montelukast (including polyvitamins, mineral supplements, analgesics, and mucolytics), who were overweight or obese (body mass index [BMI] > 85th percentile), and who had concomitant diagnosed asthma, allergic rhinitis, or atopic dermatitis were excluded from the study [20–24]. Twelve children who were diagnosed with PU, seven patients in whom an underlying cause was identified [thyroid autoantibodies in three children,* Helicobacter pylori* (*H. pylori*) infection in two patients, food allergy in one, and urinary infection in one], one patient who was obese, two patients who had a history of paternal smoking, one patient whose family refused to participate in the study, six patients who had proven concomitant asthma, four patients who had concomitant allergic rhinitis, and one patient who had concomitant atopic dermatitis were excluded from the study (Figure 1). The control group was constituted by 41 healthy children who were periodically attending pediatric welfare clinics of the same hospitals for regular checkups. Children who were included in the control group had no history of acute or chronic urticaria, no history of any allergic disease, and no paternal or maternal smoking and were not taking any medications, including multivitamins, minerals, and analgesics. These subjects also had no signs and symptoms of infectious disease and were not overweight or obese. The study was performed in accordance with the tenets of the Declaration of Helsinki and good clinical practice and was approved by the Bezmialem Vakif University Ethical Committee (Number 71306642/050-99/88). Informed consent was obtained from the parents of all study participants.

### 2.2. Assessment of Disease Activity

Disease activity was monitored using the urticaria activity score (UAS7) according to EAACI/GA^2^LEN/EDF/WAO Guidelines [10]. The UAS7 consisted of the sum of the wheal number score and the itch severity score, that is, the sum score of seven consecutive days, which can be from 0 to 42 (Table 1). The guidelines state that UAS7 is best measured by patients documenting 24-hour self-evaluation scores once daily for several days [10]. Therefore, we teach our patients and their families how to calculate the UAS7 and instruct them to bring us this information.

### 2.3. Blood Sample Collection

Antihistamines and montelukast were discontinued at least 24 hours before blood sampling. After overnight fasting, peripheral blood samples (total, 4 mL) were collected from an antecubital vein into heparinized tubes; thereafter, blood was centrifuged at 1500 ×g for 10 min to obtain the plasma. The separated plasma was then stored at −80°C until further analysis of TAS and TOS levels.

### 2.4. Laboratory Investigations

Complete blood count, erythrocyte sedimentation rate, liver function tests, thyroid stimulating hormone, free thyroxin, serum total IgE, anti-thyroid peroxidase and anti-thyroglobulin antibodies, microscopic investigation of stool for parasites and stool enzyme immunoassay for* Helicobacter pylori* antigens, urinalysis with test strips, and urine cultures were carried out in all patients as per standard laboratory procedures.

Skin prick tests and ASST were also performed. Commercial allergen solutions manufactured by Stallergenes (Paris, France) were used for skin tests. Ten different aeroallergens consisting of house dust mites, grass, tree pollens, fungi, and animal dander and nine food allergens consisting of milk, egg, wheat, soy, cocoa, peanut, hazelnut, banana, and strawberry were tested. A positive skin prick test was one with at least a wheal of maximum diameter of 3 mm once the negative value had been subtracted. ASST was performed using the method described by Sabroe et al. [25]. We were not able to perform skin prick tests and ASST in 13 patients because their antihistaminic treatments could not be discontinued due to their intense complaints caused by the disease.

### 2.5. Measurement of Total Oxidant Status (TOS)

Plasma TOS was measured using an automated method developed by Erel [26]. Oxidants present in a sample oxidize the ferrous ion of an o-dianisidine complex to ferric ions. Oxidation is enhanced by glycerol, which is abundant in the reaction medium, and the ferric ion forms a colored complex with xylenol orange under acidic conditions. Color intensity (which can be measured spectrophotometrically) is associated with the total level of oxidants present. Hydrogen peroxide is used to calibrate the assay, and results are expressed in terms of micromoles of hydrogen peroxide equivalent per liter (*μ*mol H_2_O_2_ equiv./L).

### 2.6. Measurement of Total Antioxidant Stress (TAS)

Plasma TAS was measured using another automated method developed by Erel [27]. This involves the production of the hydroxyl radical, which is a potent biological reactant. A ferrous ion solution (Reagent 1) was mixed with hydrogen peroxide (Reagent 2). Radicals produced by the hydroxyl radical, including the brown dianisidinyl radical cation, are also potent in biological terms. Thus, it is possible to measure the antioxidative capacity of a sample in terms of the inhibition of free radical reactions initiated by the production of the hydroxyl radical. Variation in assay data is very low (less than 3%), and results are expressed as mmol Trolox equiv./L.

### 2.7. Calculation of the Oxidative Stress Index (OSI)

The OSI was the TOS-to-TAS ratio. It was calculated as follows: OSI (arbitrary units) = TOS (*μ*mol H_2_O_2_/L)/TAS (mmol Trolox/L). Results are expressed as arbitrary units (AU).

### 2.8. Statistical Analyses

Statistical analysis was performed using IBM SPSS 19 (IBM, Armonk, NY, USA). The Shapiro-Wilk normality test was used to test the distribution of the data. Parametric data were expressed as the mean ± standard deviation (SD), and nonparametric data were expressed as the median, IQR (interquartile range). A Mann-Whitney *U* test was used to compare the two groups. The correlation between two variables was tested using Spearman's rho coefficient. Categorical data were evaluated using the chi square test, and a*p* value of less than 0.05 was accepted as statistically significant.

## 3. Results

The study group consisted of 37 boys and 25 girls, and the control group consisted of 22 boys and 19 girls. The mean ages of the CSU patients and those of the control group were 10.5 ± 4.1 and 9.2 ± 3.8 years, respectively. The mean values of BMI were 20.3 ± 3.7 in CSU patients and 18.9 ± 2.9 in control group. There were no significant differences between the groups with respect to age, gender, and BMI (*p* > 0.05). Forty percent of CSU patients were experiencing angioedema during their illness. The median duration of disease was 12 months (5–24) and the median value of UAS7 was 22.0 (13.0–30.5). Some clinical features of CSU patients are shown in Table 2.

**Figure 2:**
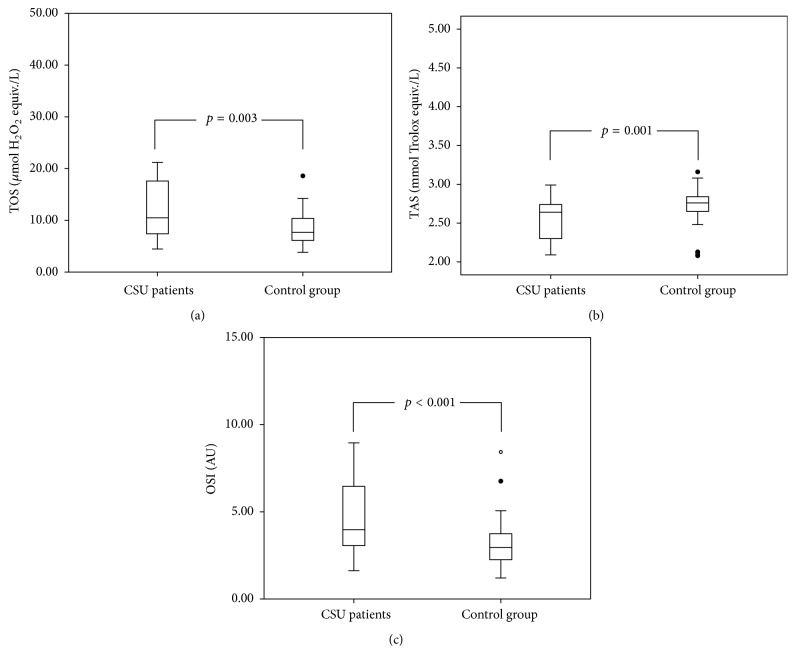
(a) Plasma total oxidant status (TOS); (b) total antioxidant status (TAS); and (c) oxidative stress index (OSI) levels in study and control groups.

**Figure 3:**
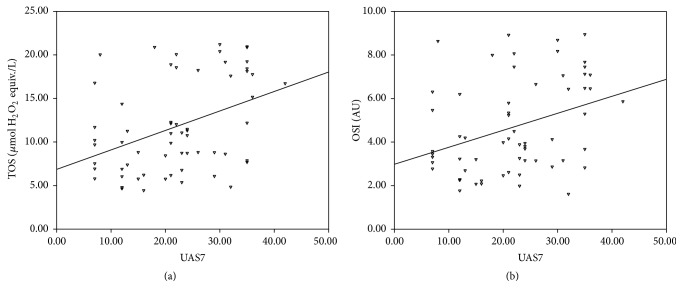
(a) Correlation graphs between total oxidant status (TOS) and urticaria activity score (UAS7) and between oxidative stress index (OSI) and UAS7 (b).

The median values of plasma TOS were 10.49 *μ*mol H_2_O_2_ equiv./L (7.29–17.65) in CSU patients and 7.68 *μ*mol H_2_O_2_ equiv./L (5.95–10.39) in the control group. The difference between the groups was statistically significant (*p* = 0.003, Figure 2). Plasma TAS levels in the CSU group were significantly decreased compared to the control group (2.64 [2.30–2.74] versus 2.76 [2.65–2.86] mmol Trolox equiv./L, resp., *p* = 0.001; Figure 2). Additionally, there was a statistically significant difference between OSI levels in the CSU group and healthy controls (3.97 [3.01–6.47] versus 2.95 [2.22–3.76] AU, resp., *p* < 0.001; Figure 2). All of these oxidative stress parameters did not vary between the groups in terms of gender, presence or absence of angioedema, or ASST positivity (*p* > 0.05). Also there was not a correlation between body weight or BMI and all these parameters (*p* > 0.05). TOS and OSI levels showed statistically significant positive correlation with UAS7 (rho = 0.381, *p* = 0.002, and rho = 0.337, *p* = 0.008, resp.; Figure 3). Moreover, TAS values showed statistically significant negative correlation with disease duration (rho = −0.407, *p* = 0.001).

## 4. Discussion

To the best of our knowledge, this is the first study showing that patients with CSU have elevated plasma TOS and OSI levels and reduced TAS levels compared to healthy controls. Also, this is the first study that focuses on the pediatric age group and demonstrates oxidative stress in CSU. This is especially important because CSU is a rare condition in children compared to adults, and recommendations for its management and treatment in the pediatric age group are based on data obtained from studies conducted on adults [28]. Even comprehensive guidelines that have been published on this issue contain little information related to children [1, 10]. We defined strict exclusion criteria to avoid confounding results. All proven diseases and factors associated with oxidative stress were eliminated as thoroughly as possible. Although only little evidence presents* H. pylori* as an etiological factor in CSU, we accepted* H. pylori* infection as an exclusion criterion for eliminating possible confusion [1, 10].

Although CSU is reportedly more prevalent in females [29, 30], there was male dominance (60%) in our study, which is compatible with studies by Volonakis et al. and Sahiner et al. [4, 31]. We did not find any difference in plasma TOS, TAS, OSI levels, and UAS7 score between boys and girls (*p* > 0.05). We excluded patients with concomitant asthma, allergic rhinitis, and atopic dermatitis from the study, because there are some studies showing that all these diseases are associated with elevated oxidative stress [22–24]. However, the frequency of concomitant allergic diseases in our CSU group was not significantly higher when compared to prevalence rates of allergic diseases in our city. The rates of elevated serum IgE, eosinophilia, and aeroallergen sensitization of our study group were compatible with literature data [31]. We concluded, like other researchers, that atopy and gender are not associated factors with CSU [31].

We detected ASST positivity in 29% of patients. Although this is consistent with the literature findings, including the results of our previous study [29, 30], higher rates of up to 46% have also been reported [31]. Sabroe et al. have suggested that the presence of a positive ASST is associated with more severe symptoms in adult patients [32], but studies conducted on children do not support this judgement and reported the same clinical severity or remission rates in children with negative ASST [30, 31, 33]. According to our results, UAS7, plasma TAS, TOS, and OSI levels were not different between ASST-positive and ASST-negative CSU patients (*p* > 0.05). Hence, AIU is not associated with more pronounced oxidative stress or more severe clinical courses compared to idiopathic forms of CSU.

There is a growing body of literature on the role of oxidative stress in allergic diseases, especially asthma, and to a lesser degree atopic dermatitis [11, 22–24]. There are only a few studies with conflicting results in urticaria [34–37]. There are many members of the oxidative and antioxidative systems, and the half-lives of ROS are usually short. Thus, the measurement of these molecules individually is time-consuming and costly and requires complicated techniques. Plasma TOS and TAS measurements are rapid, easy, reliable, sensitive, and inexpensive methods for assessing oxidative stress and have shown good correlation with other markers of oxidative stress and clinical parameters [23, 26, 27]. Although the normal values of TAS and TOS levels in healthy newborns have been determined, the normal values of other age groups including our study group are not yet known [38]. TAS and TOS values were not statistically different in male and female newborns in this study [38]. But it is well known that younger people have higher levels of oxidative stress biomarkers due to their high metabolic rates [12].

Cassano et al. reported elevated ROS production in platelets of adult CU patients compared to healthy controls, and, after treatment with desloratadine, they showed significant decreases in ROS production [34]. Nevertheless, even after treatment with desloratadine, ROS production was still high and not comparable to healthy controls [34]. In another study performed in adult females with CSU, plasma and erythrocyte manganese superoxide dismutase (MnSOD), copper-zinc superoxide dismutase (Cu/ZnSOD), glutathione peroxidase (GSH-PX), and catalase (CAT) activities were measured as indices of enzymatic antioxidant capacity, as well as malondialdehyde (MDA) levels as a marker of lipid peroxidation [35]. However, no difference was detected in the levels of those markers between the patients and healthy controls. Nonenzymatic antioxidant capacity could not be evaluated in this study [35]. It is well known that the antioxidant system has numerous members, whose antioxidant effects are additive [12, 27]. Results might also be influenced by the study population, which consists of female patients only, because the expression of some antioxidant enzymes, including those that are evaluated in their study, and others, like paraoxonase-2, is influenced by gender and sex hormones [39, 40]. Sagdic et al. reported that patients with CU have similar erythrocyte MDA levels and GSH-PX activities and decreased Cu/Zn SOD activities compared to healthy controls [36]. However, only 25 patients are enrolled in this study; hence, the small sample size makes it difficult to demonstrate the difference between the groups statistically. On the other hand, in the study of Kalkan et al., patients with acute urticaria were found to have elevated Cu/Zn SOD activities and MDA levels and decreased plasma GSH-PX activities compared to healthy controls [37].

Although UAS7 is family-derived information, it is a simple, widely accepted, and validated scoring system [10]. CSU symptoms change frequently in intensity and physicians can assess symptoms only for a short period of time. Therefore, disease activity is best measured by self-evaluation scores for several days [10]. Our findings revealed that plasma TOS and OSI levels are positively correlated with UAS7 score (*p* = 0.002 and *p* = 0.008, resp.). Likewise, in the study of Rajappa et al., the authors reported that adult patients with CSU had significantly reduced platelet SOD and GSH-PX levels and elevated platelet MDA levels compared to healthy controls. Platelet MDA levels also showed a statistically significant positive correlation with urticaria severity score [41]. We also showed a statistically significant negative correlation between the duration of CSU and plasma TAS levels (rho: −0.407, *p* = 0.001). The reduction in the plasma levels of the antioxidant system components for balance of the oxidative stress over a prolonged period may be possible explanation of this situation. However, TOS and OSI levels were not correlated with disease duration (*p* > 0.05).

Wheals, flares, and angioedema, which are characteristics of CSU, develop as a result of increased vascular permeability and extravascular leakage of intravascular fluid and proteins [42]. Also, there is convincing evidence showing that ROS can directly lead to degranulation of mast cells, endothelial dysfunction, and increased vascular permeability [11, 18, 19, 43]. When our study results and the aforementioned literature data are analyzed together, it comes plausible that antioxidants may be useful complementary therapies for CSU. We think that despite some limitations vitamin C, vitamin E, carotenoids, selenium, and N-acetylcysteine may be promising molecules for this purpose [44–51].

## 5. Conclusion

Our study results showed that oxidative stress is increased in children with CSU and is positively correlated with the disease activity. This information is valuable for both understanding the pathogenesis of CSU and finding targeted, more effective, and less toxic treatment alternatives. Fortunately, many drugs currently available, such as vitamins and minerals, can modify oxidative stress. However, for a better understanding of this issue many more studies are needed.

## Figures and Tables

**Table 1:** The UAS7 for assessing disease activity in CSU [10].

Score	Wheals	Pruritus
0	None	None
1	Mild (<20 wheals/24 h)	Mild (present but not annoying or troublesome)
2	Moderate (20–50 wheals/24 h)	Moderate (troublesome but does not interfere with normal daily activity or sleep)
3	Intense (>50 wheals/24 h or large confluent areas of wheals)	Intense (severe pruritus, which is sufficiently troublesome to interfere with normal daily activity or sleep)

**Table 2:** Some demographic and clinical features of the study group.

	CSU patients (*n* = 62)
Age years (mean ± SD)	10.5 ± 4.1
Gender (M/F)	37/25
Disease duration months (median, [IQR])	12 (5–24)
Angioedema (*n* [%])	25/62 (40%)
BMI (mean ± SD)	20.3 ± 3.7
Total IgE > 100 I*µ*/L (*n* [%])	22 (35%)
Eosinophils > 5% (*n* [%])	6 (10%)
Aeroallergen sensitization (*n* [%])	10/49 (20%)
ASST positivity (*n* [%])	
ASST-positive	14/49 (29%)
ASST-negative	35/49 (71%)
UAS7 (median, [IQR])	22.0 (13–30.5)

CSU, chronic spontaneous urticaria; SD, standard deviation; IQR, interquartile range; ASST, autologous serum skin test; UAS7, urticaria activity score; BMI, body mass index.
